# Nutritional quality of foods according to the Nova food classification system after a behavioral economics intervention in food pantries

**DOI:** 10.3389/fpubh.2025.1613200

**Published:** 2025-08-29

**Authors:** Kristen Rossi, Maria F. Gombi Vaca, Marlene B. Schwartz, Caitlin Caspi

**Affiliations:** ^1^Department of Allied Health Sciences, University of Connecticut, Storrs, CT, United States; ^2^Rudd Center for Food Policy and Health, University of Connecticut, Hartford, CT, United States; ^3^Department of Human Development and Family Sciences, University of Connecticut, Storrs, CT, United States

**Keywords:** ultra-processed foods, Nova classification system, nutritional quality, behavioral economics, food pantry, consumer behavior

## Abstract

**Introduction:**

Consumption of ultra-processed foods has been linked with poor health outcomes. Using the Nova food classification system to measure the level of food processing, this study assessed whether foods selected by food pantry clients were more favorable following a behavioral economics food pantry intervention.

**Methods:**

The study analyzed secondary data from a group-randomized evaluation in 11 Minnesota food pantries. Food items selected by 187 clients (85 intervention, 102 control) were categorized according to Nova in one of four categories: (1) unprocessed/minimally processed, (2) culinary ingredients, (3) processed foods, or (4) ultra-processed foods. In each client cart, the energy share (% of total calories) of each Nova food category was calculated. Adjusted mixed linear models were used to test the post-intervention differences in the energy share of Nova food categories between intervention conditions.

**Results:**

On average, unprocessed/minimally processed foods represented 34.6% of the energy share among carts in the intervention group and 33.8% among client carts in the control group. Ultra-processed foods represented 43.5 and 41.1% of the foods in the control and intervention groups, respectively. In the adjusted models, no statistically significant differences in the energy share of Nova categories were found between foods selected by clients in the pantries in the two conditions.

**Discussion:**

Neither a reduction in client selection of ultra-processed foods nor an increase in unprocessed/minimally processed foods were among the benefits of the intervention. Future research should explore interventions targeting Nova food categories and subgroups to improve the nutritional quality of foods in food pantries.

## 1 Introduction

According to the United States Department of Agriculture (USDA), approximately 13.5 million households in the United States were food insecure in 2021 (1). Food pantries provide food directly to families who face financial difficulty when shopping at conventional grocery stores or who live in areas where there may not be access to healthy and affordable food (2). Compared with the United States population, food pantry clients are disproportionately affected by increased risks of diet-related chronic diseases (2).

Food pantries’ constraints on sourcing perishable foods, such as fresh fruits and vegetables, include limited refrigeration and dependence on food supplied through donations, federal community food programs, and inconsistent food rescue streams. Food pantries typically source and distribute non-perishable and shelf-stable foods, including processed or ultra-processed foods (UPF). According to the Nova food classification system, foods classified as UPF (Nova 4) are industrial formulations that tend to be high in energy density, fat, sugar, and salt, usually containing multiple ingredients and food additives (3). This category of foods includes soft drinks, confectionery, savory snacks, and breakfast cereals. Other Nova categories are unprocessed or minimally processed (fruits, eggs, milk, rice, dry pasta); processed culinary ingredients (sugar, vegetable oil); and processed foods (salted or sugared fruits and nuts, smoked meats, fresh bread) (3).

A study conducted in two food pantries in Montana found that two-thirds of all calories came from either processed or UPF (4). Clients who rely on food pantries have substantial exposure to UPF since they tend to obtain most of their food from these settings (2, 5, 6). There is mounting evidence supporting the negative impact of UPFs on overall health (7–10). A 2022 systematic review and dose–response meta-analysis found that with every 10% increase in daily caloric consumption of UPF, there was an associated 15% increase in all-cause mortality (10).

To improve food pantry clients’ dietary and health outcomes, interventions in these settings have used behavioral economics to influence, or “nudge,” individual behavior in favor of healthier choices (11, 12). “Nudging” can include cues, shelf signage, and manipulation of food item displays to encourage the selection of healthier items (13, 14). Behavioral economics approaches have been shown to improve the overall healthfulness of food pantry client selections without restricting choices (15–17).

The SuperShelf intervention used organizational changes and behavioral economic strategies to improve the supply and demand for healthy food in 11 Minnesota food pantries (18). In the intervention, food pantries worked on sourcing and stocking healthier items to promote improved nutritional quality of clients’ selections, assessed by the 2015 Healthy Eating Index (HEI) (19). An increase of 6.3 points and 1.7 points were observed in average total HEI scores in the intervention and control groups, respectively. However, this was not found to be a statistically significant difference (*p* = 0.560) (18). HEI and Nova are independently important in assessing food quality and understanding its health impact. While HEI scores are often used to assess diet quality in the U. S., the Nova classification system is increasingly used in international settings, including Malaysia, Israel, Brazil, and France (20). While the purpose of the SuperShelf intervention was not to address food processing levels specifically, in making changes to improve the supply and demand for healthy food, the intervention could reduce UPF by de-emphasizing them and promoting less processed options such as fruits, vegetables, and whole grains. Therefore, this study is a secondary, exploratory analysis of the SuperShelf intervention to assess differences in the level of food processing in foods selected by food pantry clients. We hypothesized that clients in the intervention group would select fewer UPF than clients in the control group.

## 2 Methods

### 2.1 Study design overview

Sixteen food pantries were recruited via an online application process for the SuperShelf evaluation study in 2018–2019. Food pantries were eligible to apply to participate if they were located in Minnesota, offered a client choice for food selection, and had the staffing and capacity to participate in intervention and evaluation activities, which included data collection at baseline and after 1 year. Food pantries were randomized in a 1:1 ratio to an intervention (eight pantries) and delayed intervention (eight pantries) condition in which the delayed intervention group agreed not to make SuperShelf-related changes until the completion of post-intervention data collection. Due to the COVID-19 pandemic, only 11 food pantries completed all evaluation elements. This included five pantries randomized to the intervention group and six to the control group.

### 2.2 SuperShelf intervention

There were two main phases of the intervention, which were completed between February 2018 and March 2020. Phase one, which focused on supply, aimed to introduce operational strategies to promote access to healthy and culturally appropriate foods while meeting stocking standards to ensure consistency of supply in the pantry (18). Phase one was implemented over a period of 1–2 months, after which point the second phase was implemented. The second phase utilized behavioral economics strategies to “nudge” clients to make the healthiest selection. In the participating food pantries, food on the shelf was arranged into food groups (fruits and vegetables, grains, proteins, dairy, and cooking and baking items) to make healthy foods the focal point and decrease the prominence of less healthy foods. These foods were followed by highly processed foods such as mixed meals, snacks, and desserts, which were placed last. Healthy food was emphasized through strategies such as placing food at eye level and bundling several products on the shelf to make a meal. Signage displayed attractive images of healthy food, including fresh fruits and vegetables. Once implemented, phase one and two changes were sustained through the follow-up measures, which took place 1 year after baseline. Additional details about the SuperShelf intervention methods are found in a separate paper (18).

### 2.3 Client sample

Two distinctive samples of clients were recruited at participating food pantries, one at baseline and one at post-intervention (1 year after baseline). A convenience sample of food pantry clients was enrolled at baseline and follow-up. All clients were approached at the end of their food pantry visit after they had selected their food. The research team agreed on a data collection start date with the food pantry. The team then screened all clients until at least 17 clients per pantry were enrolled. Clients were eligible to participate if they were at least 18 years old, spoke English, Spanish, or Somali, had access to a phone, and were mentally capable of consenting to participation. Client characteristic data - including demographics, food pantry usage, foods selected at pantry visit, and cardiovascular health - were collected through surveys, while food pantry characteristics were collected through surveys responded to by pantry managers (18).

### 2.4 Food selected by clients

All foods selected by clients at baseline and post-intervention were recorded and entered into the Nutrition Data System for Research (NDSR) (21). Using package labels or weight of unlabeled items utilizing a scale, data collectors noted product name, brand, weight, exact count, and important nutrition information included on the packaging (e.g., reduced fat or sodium) (18). For each food item, NDSR database provides a product description along with detailed nutritional information (e.g., calories, added sugar, sodium content in 100 grams of the product) and designates one of the 135 food subgroups (e.g., beef, animal fat, baby food) according to the NDSR database.

### 2.5 Applying the Nova food classification system

For this secondary analysis of the SuperShelf intervention, foods selected by clients were categorized according to the Nova food classification system. First, each NDSR food subgroup was assigned a Nova category: unprocessed or minimally processed (Nova 1), processed culinary ingredients (Nova 2), processed foods (Nova 3), or UPF (Nova 4). The NDSR database subgroups were also assigned to one of the 37 Nova food subgroups based on previous studies that applied the Nova classification system to dietary data from the U. S. (22, 23). Because NDSR database subgroups did not always align with Nova categories, food items were manually classified as needed. For example, foods classified by NDSR as part of the “fruit juices and drinks” subgroup needed to be further classified, as 100% juices are classified as Nova 1 while flavored or sweetened fruit drinks are classified as Nova 4. Manual coding of NDSR foods into Nova subgroups relied on obtaining additional information about the product. The following hierarchy of information sources was used: (1) the detailed product description from the NDSR database, (2) the raw data file from client carts, which in most cases included the product’s brand, and (3) the ingredient lists for the specific product, using the brand’s website, the USDA FoodData Central Branded Foods Database (24), and OpenFood Facts (25). In cases where no brand or nutritional information was available, the research team created a protocol for Nova classification for the specific food type similar to other nutrition-ranking protocols (e.g., unsweetened applesauce categorized as Nova 1; applesauce sweetened with natural sweeteners categorized as Nova 3; applesauce sweetened with artificial sweeteners or with flavor added categorized as Nova 4) (22, 23).

### 2.6 Data analysis

For each client cart at baseline (*n* = 212) and at post-intervention (*n* = 187), the energy share of Nova categories and subgroups (percent of calories from each Nova category and subgroup by total calories in the cart) was obtained. Then, the mean energy share for each Nova category and subgroup was calculated by intervention group at baseline and post-intervention. A descriptive analysis of Nova subgroups was conducted based on the mean energy share of Nova subgroups at post-intervention in both the intervention and control groups.

Post-intervention differences in energy share (%kcal) of each Nova category between client carts from pantries in the intervention group and pantries in the control group were assessed using linear mixed-effects models. These models account for clients clustered within each food pantry. In the models, the dependent variable was the energy share of the Nova category, and the independent variable was the intervention group (intervention or control). Four models were tested for each Nova category: (1) an unadjusted model (no covariates); (2) an adjusted model, controlled for the baseline food pantry level mean energy share of Nova category; (3) a model additionally controlling for the following participant characteristics: age group, level of education, gender, race/ethnicity, household size, how often in the past year the client visited the food pantry, and how much of all the clients’ food was from this food pantry in last 6 months; and (4) a fully adjusted model, additionally controlling for the following food pantry characteristics: whether the pantry was located in an urban or rural area, the number of freezers/coolers in the pantry, and the weight of food served per month at the food pantry.

### 2.7 Sensitivity analysis

Due to the small sample size (187 client carts, 11 food pantries) and correlation between outcomes, a set of sensitivity analyses at the item level (*n* = 7,779) was conducted. Items were classified as UPF or not (non-Nova 4 item or Nova 4 item). Mixed-effects logistic regression was used to estimate the difference in the odds of selecting Nova 4 items between intervention and control groups, in which the dependent binary variable was the Nova 4 group (yes/no), and the independent variable was the intervention group (intervention or control). The mixed-effects models account for items clustered within clients and for clients clustered within each food pantry. The same model progression was used as in the linear regression models (i.e., unadjusted, adjusted for mean baseline client scores, additionally adjusted for client characteristics, additionally adjusted for food pantry characteristics). Statistical analyses were performed using Stata 17.

Human subject procedures were conducted in accordance with the 1964 Helsinki Declaration and its later amendments. The study was approved as protocol 1612S02201 at the University of Minnesota and H20-0076 at the University of Connecticut. Written informed consent was obtained from all individual participants.

## 3 Results

### 3.1 Descriptive analysis

Table 1 summarizes the characteristics of clients and food pantries at baseline and post-intervention by intervention group. Most clients were between 18 and 64 years old and identified as non-Hispanic white. Approximately half of the pantries in the intervention and control groups were in urban communities. Similarly, nearly half of the pantries in the intervention and control groups reported having less than six freezers/coolers. Finally, in both the intervention and control groups, the mean pounds of food served per month was nearly the same (29,000–31,000 at baseline; 35,000–38,000 at post-intervention). In each intervention group, food pantry clients’ characteristics were not statistically different between baseline and post-intervention using Pearson chi-square test (for categorical variables) and t-test (for the continuous variable).

**Table 1:** Characteristics of food pantry clients and food pantries by intervention group at baseline and post-intervention (SuperShelf, 2018–2020).

Food pantry clients’ characteristics	Baseline				Post-intervention			
	Intervention (*n* = 96)		Control (*n* = 116)		Intervention (*n* = 85)		Control (*n* = 102)	
	*n*	*(%)*	*n*	*(%)*	*n*	*(%)*	*n*	*(%)*
Gender								
Female	61	(63.5)	68	(59.6)	49	(58.3)	63	(61.8)
Male	35	(36.5)	45	(39.5)	34	(40.5)	39	(38.2)
Transgender	0	(0)	1	(0.9)	1	(1.2)	0	(0.0)
Age Group								
18 to 44 years old	39	(40.6)	43	(37.4)	36	(42.9)	44	(44.0)
45 to 64 years old	42	(43.8)	59	(51.3)	42	(50.0)	44	(44.0)
65 years or older	15	(15.6)	13	(11.3)	6	(7.1)	12	(12.0)
Race/Ethnicity								
Hispanic-Latino	11	(11.6)	9	(8.0)	13	(15.7)	10	(10.3)
Non-Hispanic Black	9	(9.5)	29	(25.9)	10	(12.1)	17	(17.5)
Non-Hispanic Native American	4	(4.2)	5	(4.5)	4	(4.8)	4	(4.1)
Non-Hispanic White	64	(67.3)	64	(57.1)	46	(55.4)	53	(54.6)
More than one race or Other^a^	7	(7.4)	5	(4.5)	10	(12.1)	13	(13.4)
Highest level of education								
Less than high school	10	(10.5)	18	(16.2)	8	(9.6)	6	(6.2)
High school or graduate equivalency degree	33	(34.7)	45	(40.5)	33	(39.8)	36	(37.1)
Some college, associate, or vocational-technical degree	34	(35.8)	41	(37.0)	33	(39.8)	46	(47.4)
Four-year college degree or higher	18	(19.0)	7	(6.3)	9	(10.8)	9	(9.3)
Frequency of food pantry visits								
Once a month or more	71	(75.5)	86	(74.8)	60	(72.3)	75	(73.5)
Less than once a month	23	(24.5)	29	(25.2)	23	(27.7)	27	(26.5)
Proportion of food obtained from food pantry								
Half or more	44	(45.8)	49	(42.6)	43	(51.8)	53	(52.5)
Less than half	52	(54.2)	66	(57.4)	40	(48.2)	48	(47.5)
Household Size, *median (IQR)*	94	2 (3)	115	2 (3)	81	3 (2)	97	2 (3)
Location								
Urban	3	(60)	3	(50)	3	(60)	3	(50)
Rural	2	(40)	3	(50)	2	(40)	3	(50)
Number of freezers/coolers								
Less than six	2	(40)	4	(66.7)	2	(40)	4	(66.7)
More than six	3	(60)	2	(33.3)	3	(60)	2	(33.3)
Pounds (lb.) of food served per month, *mean (SD)*	31,488 (29,734)		29,388 (13,902)		38,135 (35,163)		35,398 (15,810)	

^a^Participants that selected more than one of the possible responses to the self-classified racial background question, self-classified as “Native American” and “White or Caucasian”; “Native American,” “Black, African American” and “White”; “Black, African American” and “White”; or a unique participant write-in response.

**Figure 1:**
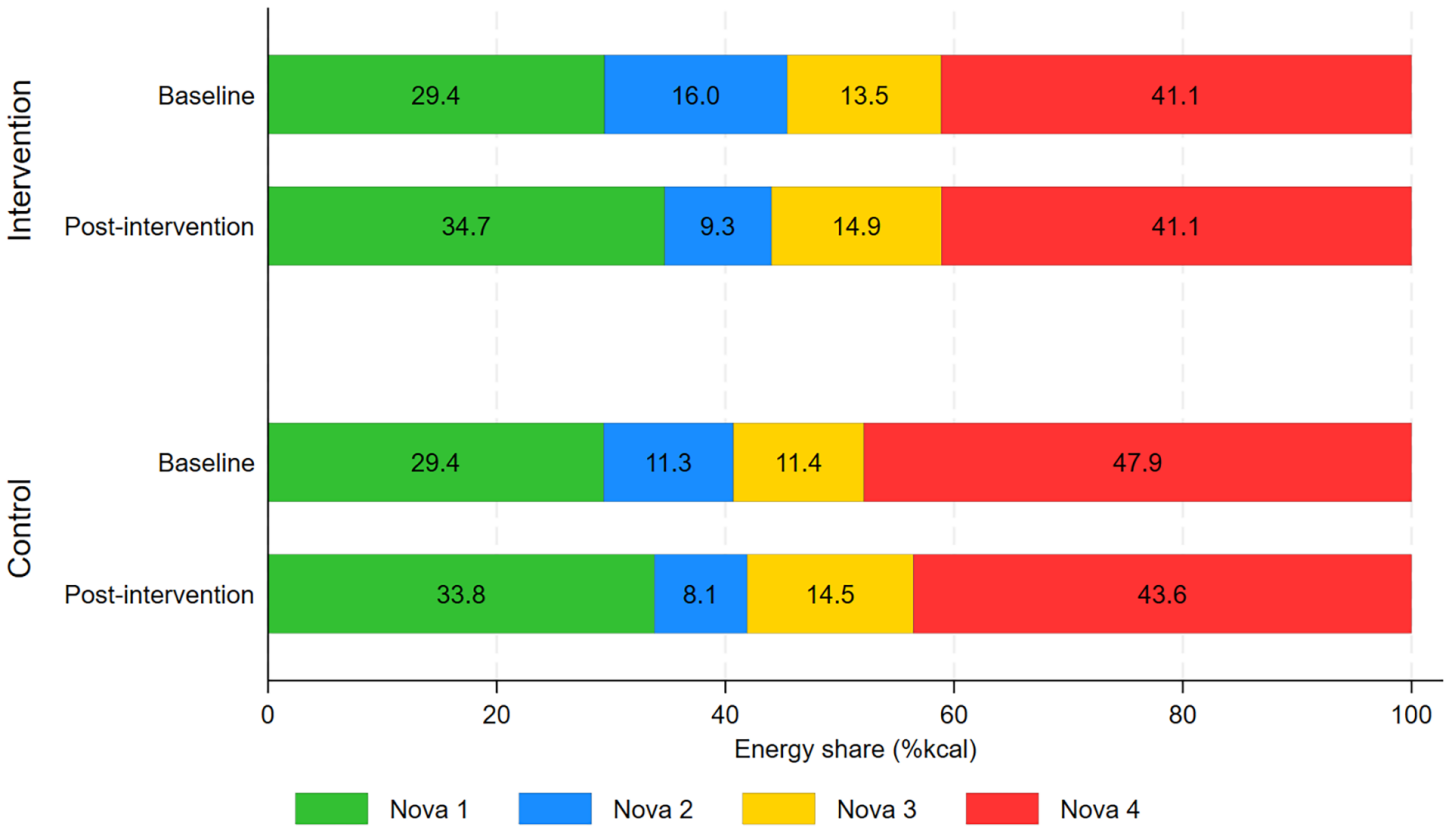
The mean energy share of each Nova category in the control and intervention groups at baseline and post-intervention (SuperShelf, 2018-2020; *n* = 399). Note. Sample sizes: Intervention, at baseline, *n* = 96, at post-intervention, *n* = 85; Control, at baseline, *n* = 116, at post-intervention, *n* = 102.

The energy share (%kcal) of each Nova category by intervention group at baseline and post-intervention is presented in Figure 1. At post-intervention, the average energy share of Nova 1 foods by calories was 34.7% in the intervention group and 33.8% in the control group. At the same time, energy from Nova 4 foods represented 41.1% in the intervention group and 43.6% in the control group. At baseline, energy from Nova 1 represented, on average, 29.4% of the calories in clients’ carts in the intervention group and 29.4% in the control group. Nova 4 represented an average of 41.1% of the calories in clients’ carts in the intervention group and 47.9% in the control group.

**Figure 2:**
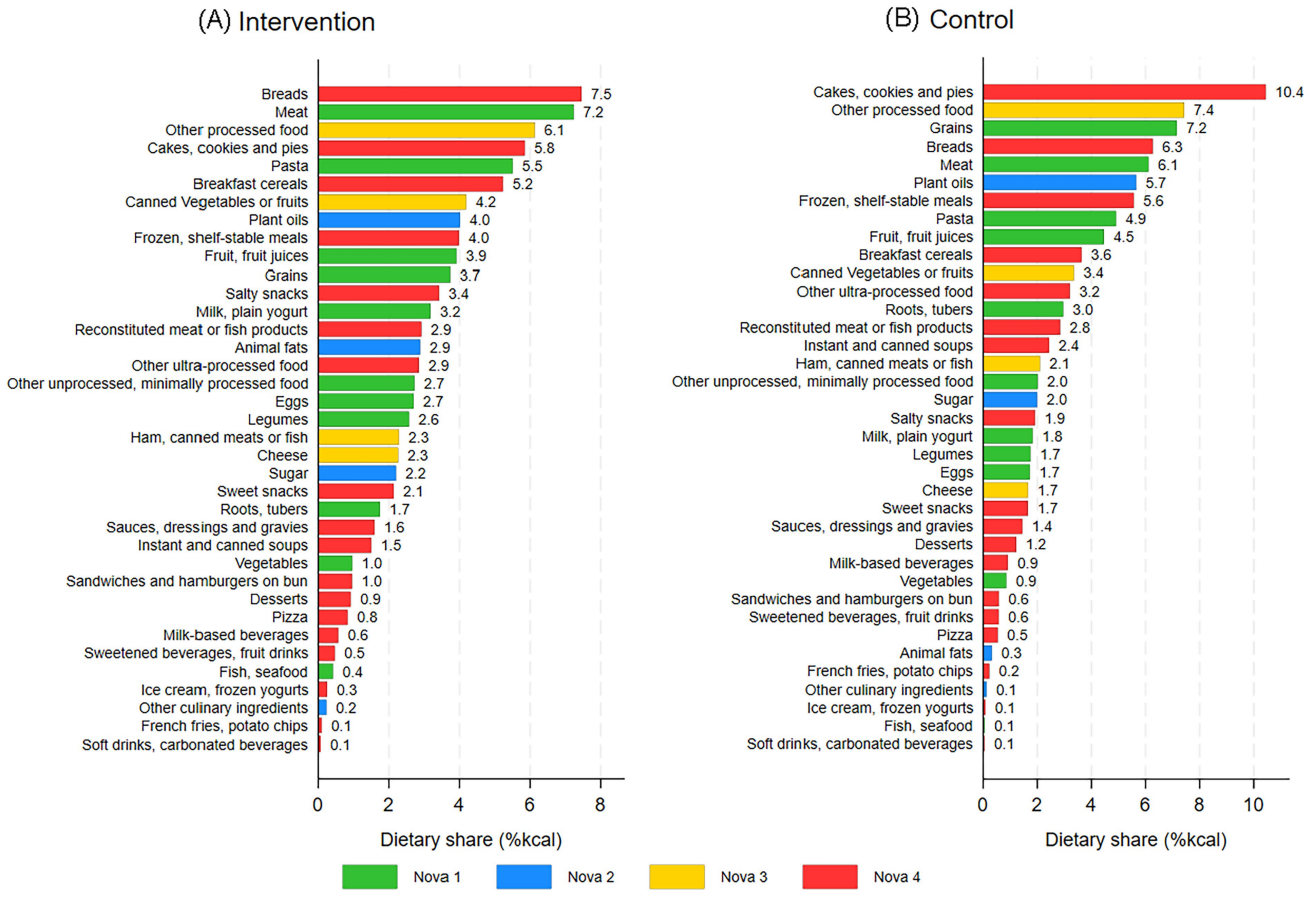
The mean energy share of Nova subgroups in the intervention group (**A**; *n* = 85) and in the control group (**B**; *n* = 102) at post-intervention (SuperShelf, 2019-2020).

Figure 2 presents the energy share of Nova subgroups in client carts in the intervention (Figure 2A) and control groups (Figure 2B) at post-intervention. Bread, meats (including poultry), other processed foods, and cakes, cookies and pies were among the highest ranked Nova subgroups by energy share (%kcal) at post-intervention for both the intervention and control groups.

### 3.2 Linear regression model results

The results of the unadjusted and fully adjusted mixed models assessing the differences in energy share (%kcal) of Nova categories are presented in Table 2. There were no statistically significant differences in the energy share of Nova categories between the intervention and control groups in the unadjusted models or any adjusted models. In the fully adjusted models, the difference in the energy share of Nova 1 was 1.68 (*p* = 0.437), Nova 2 was −0.78 (*p* = 0.761), Nova 3 was 1.73 (*p* = 0.390), and Nova 4 was 1.15 (*p* = 0.780).

**Table 2:** Results from the linear mixed effects models assessing the differences in the energy share of each Nova category between intervention and control groups at post-intervention (SuperShelf, 2019–2020; *n* = 187).

Nova category	Energy share					
	Unadjusted model			Fully adjusted model^a^		
	Estimate	SE	*p*-value	Estimate	SE	*p*-value
Nova 1	0.89	3.41	0.795	1.68	2.17	0.437
Nova 2	1.24	4.55	0.785	−0.78	2.56	0.761
Nova 3	0.33	1.73	0.847	1.73	2.02	0.390
Nova 4	−2.46	6.03	0.683	1.15	4.11	0.780

^a^Fully adjusted model was controlled for baseline food pantry mean energy share of Nova category, participant characteristics (age group, level of education, gender, race/ethnicity, household size, how often in the past year the client visited the food pantry, and how much of all the clients’ food was from this food pantry in last 6 months), and food pantry characteristics (whether the pantry was located in an urban or rural area, the number of freezers/coolers in the pantry, and the pounds of food served per month at the food pantry).

### 3.3 Sensitivity analysis results

At post-intervention, among the total number of items in the client carts in the intervention group pantries (*n* = 3,695) and in the control group pantries (*n* = 4,084), 41.8% and 42.9% were categorized as Nova 4, respectively. In the logistic mixed models, there was no significant difference in the odds of selecting Nova 4 between the intervention and control groups in the unadjusted models (OR = 0.92, *p* = 0.596), nor in the fully adjusted models (OR = 1.01, *p* = 0.895).

## 4 Discussion

The findings of this secondary analysis did not support the hypothesis that an intervention designed to emphasize the supply and demand of healthier food categories and de-emphasize less healthy food categories would result in a more favorable set of foods as measured by the Nova classification system. Instead, compared with a control condition, clients who selected foods at a SuperShelf intervention pantry were no less likely to select UPF (Nova 4), nor were they more likely to select Nova 1 foods (unprocessed or minimally processed).

Descriptively, there was a decrease in Nova 2 (culinary ingredients) between baseline and post-intervention in the intervention arm. This might be explained by the fact that two different samples were compared. It also may be that clients who select culinary ingredients at one visit do not need to get the same ingredients again for several weeks or months. Indeed, many clients did not report the selection of any Nova 2 foods. The analysis of food subgroups (Figure 2) aimed to understand food pantry clients’ selections better. This knowledge can guide food groups to prioritize future public health interventions in food pantries.

The results of this analysis are consistent with the overall SuperShelf findings that showed no statistically significant differences by intervention condition in the diet quality of the food selected by clients as measured by the HEI (18). SuperShelf environmental changes implemented in the intervention group had high fidelity (26), but these strategies were not focused on the processing levels of foods, which limits their impact on improving the selection of foods based on processing degree. To the author’s knowledge, only one intervention in the charitable food system to date has specifically aimed to address UPF. In that study, the UnProcessed Pantry Project (UP3), food pantry clients demonstrated improvements in diet quality following the intervention, but clients were not assessed for changes in the level of food processing of the foods they selected or consumed (27). In settings outside of the charitable food systems, interventions addressing UPF consumption have demonstrated promising dietary outcomes among adults (28, 29). Taken together, these studies suggest that interventions built around the Nova food classification system can potentially result in improvements in diet quality, including interventions based in food pantries, but more research is needed. Future intervention strategies to promote healthier food selection in food pantries may include emphasizing Nova 1 foods and deemphasizing Nova 4 foods, educational materials targeting the level of processing in foods, and increasing the availability of culturally connected foods that can be part of healthy meals. As with any public health recommendation, however, it is essential to consider the risk of inadvertently labeling some foods usually prioritized in charitable food settings (30). For example, products considered a nutrient-dense alternative, such as fortified whole grain bread, and some culturally relevant foods for food pantry clients, such as mixed dishes, dressings, and sauces, may be classified as UPF. These types of UPF, however, comprise a minority of this food group. Another possible caveat is that, for food pantry clients, who may have limited time, resources, or skills to prepare all food from scratch, UPF’s convenience may be a desirable feature that is balanced against other factors (4, 6). Therefore, it is essential to critically examine which UPF subgroups, based on their purposes (e.g., being selected as a healthier alternative or used as part of a culturally relevant meal), may not be deemphasized in the already challenging food pantry setting.

This study has several other limitations. First, while the SuperShelf intervention had the potential to reduce UPF selection, given its focus on healthy foods, it was not explicitly designed with this aim. Second, the study examined the level of UPF in clients’ food selection, which is a proxy but not a measure for clients’ food consumption, especially when food from food pantries is not their only food source. Third, the types of food available in pantries at any given time are highly variable, given the pantries’ dependence on resources and donations. This can potentially impact the food items available at both baseline and post-intervention. Finally, as data were not collected on the same set of clients at baseline and post-intervention, the analysis could not determine whether the intervention promoted changes in food selection within individuals or whether post-intervention comparison findings resulted from residual confounding by individual or pantry characteristics or contextual factors, limiting causal inference.

To address these limitations, future studies should carefully control for fluctuations in pantry inventory and food distribution over time. The incorporation of longitudinal data on food supply and client selections could strengthen causal inference. Additionally, to further address UPF consumption among clients, interventions within the food pantry environment could be paired with complementary interventions that promote less processed food consumption, such mobile applications to reduce food waste (31), or situating food pantries near produce markets.

## 5 Conclusion

Neither a reduction in client selection of Nova 4 foods nor an increase in Nova 1 foods were among the benefits of the SuperShelf intervention. An intervention specifically focusing on Nova categories and subgroups within food pantries would be worthwhile exploring, as UPF (Nova 4) generally have a poor nutritional profile and unprocessed or minimally processed foods (Nova 1) are typically more nutrient-dense. Moreover, multicomponent interventions, such as including a nutrition education component and environmental changes in food pantries, may also help promote increased adherence to positive behaviors. Promoting healthy food in the food pantry setting is crucial to mitigate clients’ diet-related health risks.

## Data Availability

The data analyzed in this study is subject to the following licenses/restrictions: deidentified data from this study are not available in a public archive but will be made available upon reasonable request. Requests to access these datasets should be directed to Caitlin Caspi, caitlin.caspi@uconn.edu.
